# Constituents of *Cryptotaenia japonica* Inhibit Melanogenesis via CREB- and MAPK-Associated Signaling Pathways in Murine B16 Melanoma Cells

**DOI:** 10.3390/molecules21101296

**Published:** 2016-09-28

**Authors:** Zuh-Kyung Seong, Sung-Yoon Lee, Amrit Poudel, Sei-Ryang Oh, Hyeong-Kyu Lee

**Affiliations:** 1 Natural Medicine Research Center, Korea Research Institute of Bioscience & Biotechnology, Yeongudanji-ro 30, Ochang-eup, Cheongwon-gu, Cheongju-si 28116, Korea; sls0486@kribb.re.kr (Z.-K.S.); yooni7@kribb.re.kr (S.-Y.L.); amrit@kribb.re.kr (A.P.); seiryang@kribb.re.kr (S.-R.O.); 2 Biomolecular Science, University of Science & Technology, 217 Gajeong-roYuseong-gu, Daejeon 34113, Korea

**Keywords:** *Cryptotaenia japonica*, murine B16 melanoma cells, flavonoids, anti-melanogenic effect, MEK, hyperpigmentation

## Abstract

Melanin plays an important role in protecting the skin against ultraviolet light and is responsible for skin color. However, overproduction of melanin is related to several skin disorders, such as age spots, freckles, café au lait spots, Becker’s nevus and other hyperpigmentation syndromes. The aim of this study was to identify the effects of kaempferol-7-*O*-β-d-glucuronide (K7G) and tilianin, isolated from *Cryptotaenia japonica*, on melanogenesis and their mechanisms of action in murine B16 melanoma cells. The α-melanocyte-stimulating hormone (α-MSH)-induced melanin production was significantly inhibited by K7G and tilianin in a dose-dependent manner. The effects of these compounds on the signaling pathway of melanogenesis were examined. K7G and tilianin downregulated the expression of microphthalmia-associated transcription factor (MITF) and melanocyte-specific enzymes, i.e., tyrosinase and TRP1. These compounds also inhibited the phosphorylation of cyclic adenosine monophosphate (cAMP)-response element binding protein (CREB) in a dose-dependent manner. In addition, these compounds increased the phosphorylation of extracellular signal-regulated kinase (ERK) but decreased the phosphorylation of c-Jun N-terminal kinase (JNK) in B16 cells. Based on the above results, the anti-melanogenic effects of these compounds are caused by suppression of the MAPK signaling pathway through the down-regulation of α-MSH-induced CREB accumulation. This finding suggests that K7G and tilianin may be good candidates for further research to develop therapeutic agents for hyperpigmentation diseases.

## 1. Introduction

Melanocytes are located at the epidermal-dermal junction and produce pigment granules, i.e., eumelanin and pheomelanin, which are responsible for the coloration of skin and hair [1]. Although melanin plays an important role in the protection of skin against ultraviolet (UV) light, abnormal changes in melanin, such as overproduction and accumulation of pigments, may be caused by skin irritation, hormonal changes, aging, a metabolic disorder, or other underlying problems. Melasma, age spots, freckles, café au lait spots, Becker’s nevus, macules due to Peutz-Jeghers syndrome and lentigos are examples of hyperpigmentation syndromes [2,3,4]. These abnormal changes greatly influence the quality of life.

Melanin is synthesized by melanocyte-specific enzymes, such as tyrosinase and tyrosinase-related protein 1 [1]. TRP-1 forms a complex with the enzyme to induce melanin formation in murine melanocytes. Microphthalmia-associated transcription factor (MITF) is a key transcription factor in melanogenic processes [5,6,7] that can transcriptionally regulate the expression of these three enzymes. The intracellular cyclic adenosine monophosphate (cAMP)-mediated pathway is a major signaling pathway that induces melanogenesis in melanocytes. cAMP increases the expression of MITF through activation of the cAMP-response element binding protein (CREB) transcription factor [8]. 

Furthermore, members of the mitogen-activated protein kinase (MAPK) family, which consists of extracellular signal-regulated kinase (ERK), c-Jun N-terminal kinase (JNK), and p38 MAPK, are known to be activated by various extracellular stimuli [9,10]. JNK and p38 kinase are stimulated by proinflammatory cytokines and environmentally induced stresses such as UV irradiation, heat and DNA damage. Phosphorylated p38 and JNK can activate MITF. In contrast, phosphorylation of ERK can reduce melanin synthesis by suppressing MITF expression [11,12]. 

Chemical agents such as hydroquinone, arbutin, kojic acid and phenylthiourea have been utilized for treatment of pigment disorders but did not satisfy patients due to their adverse effects and low efficacy [13]. Consequently, developing more effective materials for the regulation of human skin pigmentation has been a longstanding goal in the areas of pharmaceutical and cosmetic applications [6].

*Cryptotaenia japonica* (Apiaceae) is a perennial plant that is widely distributed at moist and shady spots in Korea, China and Japan [14]. The leaves of *C. japonica* have been used for food as a green vegetable and to treat lung inflammation, herpes zoster, skin itching and meningitis in folk medicine. However, this plant is occasionally confused with *Pimpinella brachycarpa* due to a similar leaf shape [15,16,17]. A few scientific reports have described essential oils and flavonoids with anti-oxidative and anti-inflammatory effects from *C. japonica* [1,18,19]. We have been searching for candidates from plants that can regulate skin pigmentation problems. Oxidative stress-mediated disorders, some diseases such as Peutz-Jeghers syndrome and other various environmental factors frequently induce hyperpigmentation [3]. This paper reports new active compounds with anti-melanogenic activity from the leaves of *C. japonica*. 

## 2. Results and Discussion

### 2.1. Effect on Melanin Content and Cytotoxicity of Compounds Isolated from the Leaves of C. japonica

**Figure 1:**
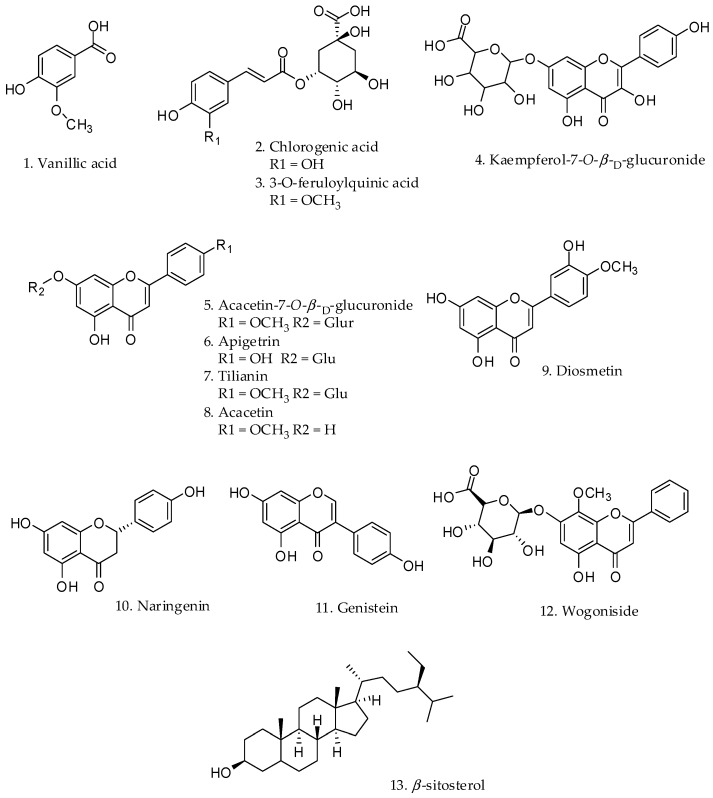
Structures of the compounds isolated from *C. japonica*.

A MeOH extract of *C. japonica* was suspended in water and partitioned with hexane. The aqueous fraction was subjected to Diaion HP-20 column chromatography with gradient elution using an increasing concentration of MeOH in water to yield six fractions (denoted CJWH1 to CJWH6) based on C_18_-TLC (thin-layer chromatography) results. Among the fractions, CJWH5 contained the major constituents of the aqueous fraction. Several types of chromatography of CJWH5 led to the isolation of the following twelve compounds, with Compound **13** isolated from the hexane fraction: vanillic acid (**1**), chlorogenic acid (3-*O*-caffeoylquinic acid) (**2**), 3-*O*-feruloylquinic acid (**3**), kaempferol-7-*O*-β-d-glucuronide (**4**), acacetin-7-*O*-β-d-glucuronide (**5**), apigetrin (**6**), tilianin (**7**), acacetin (**8**), diosmetin (**9**), naringenin (**10**), genistein (**11**), wogoniside (**12**) and β-sitosterol (**13**) (Figure 1) [20,21,22,23,24,25,26].

**Figure 2:**
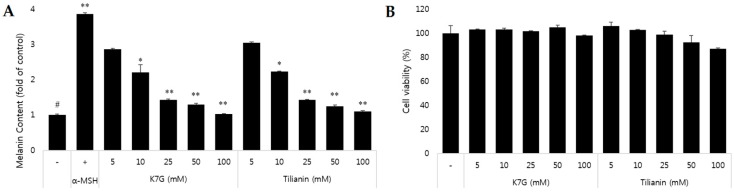
Inhibitory effect of melanogenesis of kaempferol-7-*O*-β-d-glucuronide (K7G) and tilianin in high concentration and cell viability in B16F10 cells. The cells were treated with the samples and stimulated by α-MSH (200 nM) for 72 h. The melanin inhibition (**A**) was determined as fold of content; Cell viability (**B**) was reported as percentages. The data were expressed as the mean ± S.D. from three independent experiments performed in triplicate. ^#^ *p* < 0.0001 in comparison with the control group and * *p* < 0.005, ** *p* < 0.001 in comparison with the α-MSH group. “+” is denoted as α-MSH treated group, “-” is denoted as α-MSH untreated group.

To investigate the anti-melanogenic effects of the compounds isolated from *C. japonica*, alteration of the melanin contents in B16F10 cells by the compounds was measured first. B16 melanoma is a murine tumor cell line used for research as a model for human skin [27]. There are many literatures mentioning the comparative study between B16F10 murine melanoma cells and Human melanoma cells [28]. They showed similar gene expression and previous reports had showed similarities on protein expression of both cell lines. As shown in Table 1, K7G and tilianin of *C. japonica* showed good inhibition of melanin production in a dose-dependent manner. Cell viability was assessed using the 3-(4,5-dimethylthiazol-2-yl)-2,5-diphenyl-thiazolium bromide (MTT) assay to avoid the possibility of decreased melanin due to cytotoxicity. The cytotoxicity of K7G and tilianin with higher concentration was determined and the result showed that there was no cytotoxicity caused by active compounds (Figure 2). The half-maximal inhibitory concentrations (IC_50_) of the two compounds were 12.3 ± 3.2 μM and 15.5 ± 4.5 μM, indicating that these compounds are much more active than arbutin (149.3 ± 2.02 μM), a reference compound.

### 2.2. Effects of K7G and Tilianin on the Expression of Melanogenesis-Related Proteins

**Figure 3:**
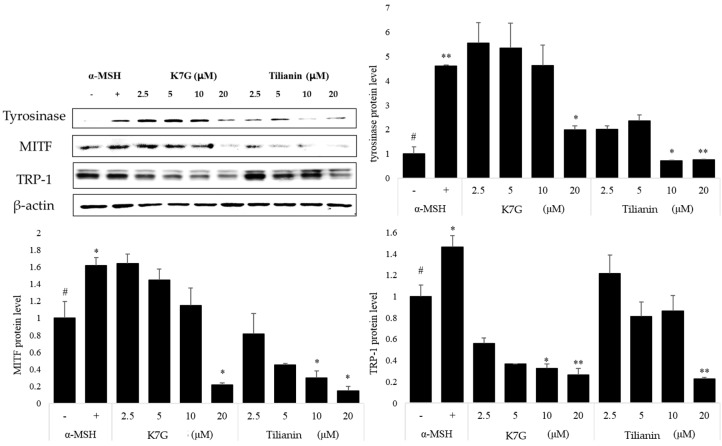
Effect of K7G and tilianin on the expression of melanogenesis-related proteins in B16F10 cells. The cells were pretreated with the samples before stimulation using α-MSH (200 nM) for 1 h and incubated for 48 h. The expression levels of the tyrosinase, TRP-1 and MITF proteins were examined by western blot analysis, as described in the experimental section. ^#^ *p* < 0.01 in comparison with the control group and * *p* < 0.05, ** *p* < 0.01 in comparison with β-actin. “+” is denoted as α-MSH treated group, “-” is denoted as α-MSH untreated group.

The effects of K7G and tilianin on the expression of tyrosinase, TRP-1 and MITF were examined by western blot analysis. The cells were treated with K7G and tilianin, separately, and then stimulated by α-MSH for 48 h. The time interval for the measurement followed that in previous studies [5]. Tyrosinase and MITF proteins were significantly suppressed by K7G and tilianin (Figure 3). MITF is a key transcription factor for regulating the expression of melanogenesis-related genes [29]. These two compounds also suppressed the protein expression of tyrosinase and TRP-1. These results indicate that the anti-melanogenic effects of K7G and tilianin result from the down-regulation of MITF expression.

### 2.3. Effects of K7G and Tilianin on CREB Phosphorylation

**Figure 4:**
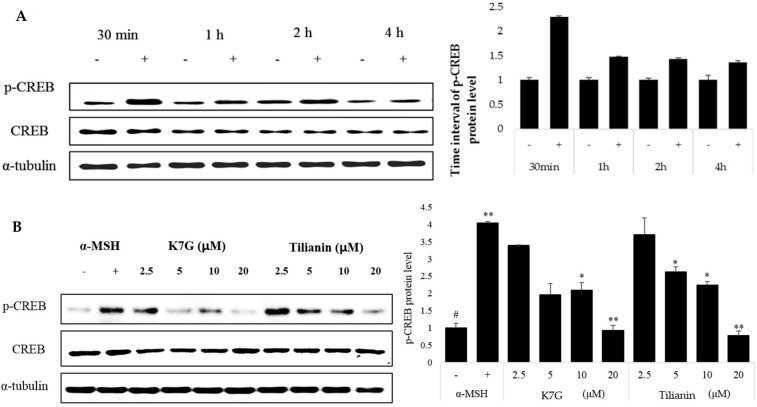
Effect of K7G and tilianin on the suppression of cyclic adenosine monophosphate (cAMP)-response element binding protein (CREB) phosphorylation in B16F10 cells. (**A**) Cells were activated by α-MSH at different time intervals; (**B**) Cells were pretreated with the test samples for 1 h before stimulation by α-MSH (200 nM) and incubated for 1 h. The expression levels of the CREB proteins were determined by western blot analysis. ^#^ *p* < 0.01 in comparison with the control group and * *p* < 0.05, ** *p* < 0.01 in comparison with α-tubulin. “+” is denoted as α-MSH treated group, “-” is denoted as α-MSH untreated group.

The cAMP-mediated signaling pathways have major roles in the regulation of skin pigmentation [30]. Specifically, cAMP activates CREB, which leads to upregulation of MITF expression. The optimized reaction time for CREB was determined by activating cells with only α-MSH (Figure 4A). The cells were treated with K7G and tilianin for 1 h, respectively, and then stimulated by α-MSH for 1 h. As observed in Figure 4, CREB phosphorylation was significantly suppressed by K7G and tilianin. The regulation of α-MSH-induced CREB phosphorylation is known to be potentially important in regulating pigmentation. According to this experiment, the degree of CREB phosphorylation was reduced by K7G and tilianin in a dose-dependent manner (Figure 4B). Finally, these results indicate that the anti-melanogenic activities of the two compounds derive from the down-regulation of MITF, tyrosinase and TRP-1 through the down-regulation of CREB phosphorylation.

### 2.4. Effect on the MAP Kinase Signaling Pathway

**Figure 5:**
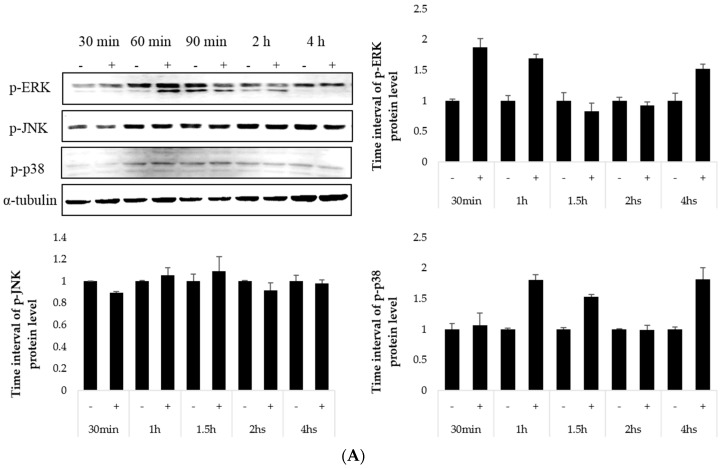

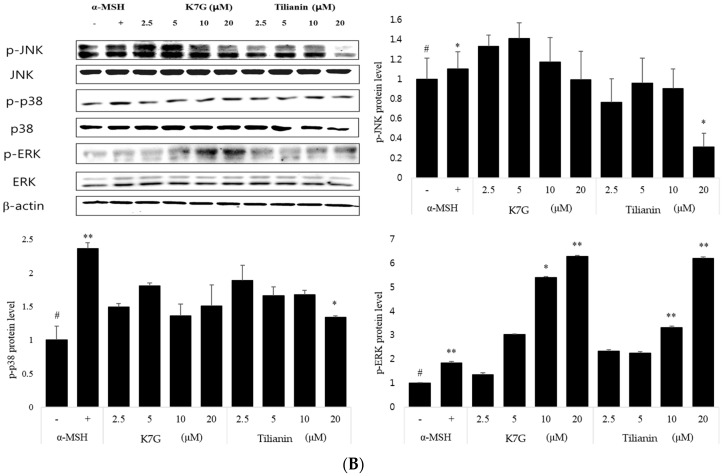
Effect of K7G and tilianin on the expression of mitogen-activated protein kinase (MAPK) proteins in B16F10 cells. (**A**) Cells were activated by α-MSH at different time intervals; (**B**) Cells were pretreated with the samples before stimulation using α-MSH (200 nM) for 1 h and incubated for 1 h. The expression levels of the MAPK proteins were examined by western blot analysis. ^#^ *p* < 0.01 in comparison with the control group and * *p* < 0.05, ** *p* < 0.01 in comparison with β-actin. “+” is denoted as α-MSH treated group, “-” is denoted as α-MSH untreated group.

To investigate if the MAPK signaling pathway is involved in the inhibitory effect of K7G and tilianin on melanogenesis, B16F10 cells were treated with two compounds at various concentrations for 1 h and then stimulated by α-MSH for 1 h. The optimized reaction time for CREB was determined by activating cells with only α-MSH (Figure 5A). K7G and tilianin enhanced the phosphorylation of ERK. On the other hand, the expression of p38 was not affected, and the phosphorylation of JNK appeared to be inhibited in tilianin**-**treated cells (Figure 5B). Several reports have indicated that the MAP kinase family members, including ERK, JNK and p38, are known to be activated by various extracellular stimuli [31]. UV radiation is one such typical extracellular stimulus that induces MAPK activation. Moreover, recent reports have implicated MAPKs in mammalian melanogenesis [32]. Previous studies reported that the downregulation of ERK signaling pathway activates cAMP-induced melanogenesis in B16 melanoma cells, which means that the increase in ERK phosphorylation may decrease melanin synthesis [33]. As expected, K7G and tilianin increased the phosphorylation of ERK, and the resulting melanin synthesis decreased, although the phosphorylation of JNK was significantly decreased by tilianin only at a high dose. 

Comparing the structures of K7G and tilianin, the skeleton (flavonol vs. flavone) and sugar moiety (glucuronic acid vs. glucose) are different, and they have a methoxyl group at the C-4′ position. These structural differences might be responsible for the variation in the phosphorylation of JNK and other activity potencies. Although the anti-melanogenic activity of K7G and tilianin and their mechanisms of action were discovered in this study, further research on the pharmacological efficacy of these compounds should be performed to develop an anti-melanogenic drug.

## 3. Materials and Methods 

### 3.1. General Experimental Procedure

The NMR spectra were recorded on a Varian UNITY 400 FT-NMR spectrometer (Varian Inc., Palo Alto, CA, USA) using tetramethylsilane as an internal standard. HR-ESI and EI mass spectra were measured using a Waters Q-ToF Premier spectrometer (Micromass UK limited, Manchester, UK) and a JEOL JMS 700 spectrometer (JEOL, Tokyo, Japan), respectively. TLC was performed on precoated silica gel 60 F_254_ and silica gel 60 RP-18F_254_ (0.25 mm, Merck, Darmstadt, Germany), and the spots were visualized by using UV irradiation (254 nm) and spraying the plates with a 10% H_2_SO_4_ solution, followed by heating. Column chromatography was performed over Sephadex LH-20 (25–100 μm, Sigma-Aldrich, Steinheim, Germany), silica gel (230–400 mesh, SiliCycle Inc., Quebec City, QC, Canada), RP-C18 (Cosmosil 75 C_18_-PREP, Kyoto, Japan), and Diaion HP-20 (Mitsubishi Chemical Corporation, Tokyo, Japan). Medium-pressure liquid chromatography (MPLC) was performed on a column (plastic-coated glass, 5 cm × 49 cm, Büchi, Flawil, Switzerland) with a ceramic pump, VSP 3050 (Rikakikai Co. Ltd., Tokyo, Japan). HPLC was performed with an Infinity system, model 1260, by Agilent Technologies (Santa Clara, CA, USA) equipped with a 1260 degasser, 1260 bin pump VL, 1260 ALS, 1260 TCC, and 1260 DAD. The analytical columns were an Atlantis T3, 5 µM, 250 × 4.6 mm, and a Phenomenex synergy RP-Polar-5 µM, 250 mm × 4.6 mm. Preparative LC was performed with a Gilson PLC 2020 system (Gilson Inc., Middleton, WI, USA) with Atlantis T3, 5 µM, 250 mm × 19 mm, and Phenomenex synergy RP-Polar-5 µM, 250 mm × 19 mm, columns. The MTT assay was measured in a Benchmark Microplate Reader (Bio-Rad, Hercules, CA, USA). The following reagents were obtained from the indicated companies. Solvents for extraction and isolation (methanol, acetonitrile, etc.) were purchased from the Duk-san Chemical Co. (Ansan-si, Korea) and Wintech (Gwangju-si, Korea). The LC-MS solvents (acetonitrile for LC-MS, CHROMASOLV^®^) were purchased from Fluka Analytical (Durban, KZN, South Africa). The solvent for NMR (CD_3_OD) was purchased from CellBio (Seongnam-si, Korea).

### 3.2. Plant Material

The aerial parts of *C. japonica* were collected in Namyang-ju, Kyungki Province, Korea, in August 2013. A voucher specimen (KRIB0071766) was deposited at the herbarium of the Korea Research Institute of Bioscience and Biotechnology.

### 3.3. Extraction and Isolation from the Leaves of C. japonica

Air-dried aerial parts of *C. japonica* (1.2 kg) were extracted with MeOH (6 L) at room temperature three times to obtain 329.3 g of a solid extract. The MeOH extract (329.3 g) was suspended in distilled water and then partitioned with hexane to give 84.4 g of a hexane-soluble extract and 204.1 g of an aqueous extract. The aqueous fraction (204.1 g) was applied to a Diaion HP-20 column (15 cm × 30 cm, 2 kg of HP-20) and eluted with a stepwise gradient of MeOH in distilled water (0%, 50%, 80% and 100% MeOH, each 16 L) to yield 6 fractions (denoted CJWH1 to CJWH6). Using TLC, CJWH5 (11.0 g) was shown to contain the major components. This fraction was combined and subjected to MPLC (C18-silica gel, 9 cm × 50 cm) using a stepwise gradient of MeOH in water (10%, 20%, 30%, 50% and 100% MeOH, each 8 L), yielding 23 subfractions (denoted CJWH5R1 to CJWH5R23 and the MeOH washing fraction (=CJWH5RW)). Compound **1** (16.5 mg) was obtained from fraction CJWH5R4 (820.2 mg) using LH-20 column (3 cm × 150 cm) chromatography with 90% MeOH. Compound **2** (23.5 mg) was separated from fraction CJWH5R6 (524 mg) using NP open-column chromatography (5 cm × 45 cm; 300 g of silica gel; hexane:EtOAc at 100:0, 20:1, 10:1, 5:1, 1:1). Compound **3** (21.2 mg) was obtained from fraction CJWH5R7 (2.31 g) via PLC (10%–25% acetonitrile, gradient, Phenomenex Synergi polar-RP). Compound **4** (223.2 mg) was obtained as a crystal from the CJWH5R5 fraction and removed via filtration. Compound **5** (16.5 mg) was obtained from fraction CJWH5R8 (1.31 g) using LH-20 column (3 cm × 150 cm) chromatography with 90% MeOH. The CJWH5R9 fraction was loaded on the PLC (20%–30% acetonitrile, gradient, Phenomenex Synergi polar-RP), and compounds **6** (10.2 mg), **7** (24.3 mg), **8** (4.2 mg) and **9** (16.4 mg) were isolated. Compound **10** (32.5 mg) was obtained from fraction CJWH5R11 (1.31 g) using LH-20 column (3 cm × 150 cm) chromatography with 90% MeOH. CJWH5R22 (1.93 g) was applied to MPLC, (6.5 cm × 70 cm, 700 g of C18-silica gel, MeOH:D.W., started with 100% D.W.), leading to the isolation of Compounds **11** and **12** (22.1 mg and 12.4 mg, respectively). Compound **13** (2.6 g) was separated from the hexane fraction (21.5 g) using NP open column chromatography (5 cm × 45 cm; 300 g of silica gel; Hexane:EtOAc at 100:0, 20:1, 10:1, 5:1, and 1:1).

### 3.4. Cell Culture

The murine B16F10 cells, derived from murine melanoma cells, were obtained from the American Type Culture Collection (ATCC; Rockville, MD, USA). The cells were maintained in Dulbecco’s modified Eagle’s medium (DMEM) supplemented with glutamine (1 mM), 10% heat-inactivated fetal bovine serum (FBS), penicillin (50 U/mL) and streptomycin (50 μg/mL) and incubated at 37 °C in a humidified, 5% CO_2_ incubator. For the stimulation, the medium was replaced with DMEM, and the cells were then stimulated with α-MSH in the presence of the experimental compounds for the indicated periods.

### 3.5. Cytotoxicity Assay

The cell viability was examined using the MTT assay. The murine B16F10 cells were seeded at densities of 2 × 10^4^ cells/well in 96-well plates (Thurmo Scientific, Roskilde, Denmark). The compounds were added to each well and incubated for 48 h at 37 °C in 5% CO_2_. The MTT solution (5 μL of 5 mg/mL) was added to each well, and the cells were cultured for another 4 h. The supernatant was discarded, and 100 μL of dimethyl sulfoxide (DMSO) was added to each well. The optical density of formazan was measured using a microplate reader (VeersaMax, Molecular Devices, Sunnyval, CA, USA) at 590 nm. The level of formazan generated by the untreated cells was chosen as the 100% value.

### 3.6. Determination of the Extracellular Melanin Content 

Extracellular melanin release was measured as described previously [5]. In brief, B16F10 cells were seeded in a 96-well plate at a density of 2 × 10^4^ cells/well. The next day, the cells were pretreated with the compounds (5, 10 and 20 µM) isolated from *C. japonica* for 1 h and then stimulated with 200 nM α-MSH for 72 h. The supernatant was collected. The relative melanin content was determined by measuring the absorbance at 405 nm using a microplate reader.

### 3.7. Western Blot Analysis

To determine the amount of melanogenesis-related proteins, Western blot analysis was performed. To estimate the amount of MITF, tyrosinase, and TRP-1, the cells were cultured in 60 mm dishes and pretreated with K7G and tilianin (2.5, 5, 10 and 20 µM, each); after 1 h, the cells were stimulated using α-MSH (200 nM) and then incubated for the next 48 h. To estimate the amount of ERK, JNK, and p38, the cells were cultured in 60 mm dishes and pretreated with K7G and tilianin (2.5, 5, 10 and 20 µM, each); after 1 h, the cells were stimulated using α-MSH (200 nM) and then incubated for the next 1 h. To estimate the amount of CREB, the cells were cultured in 60 mm dishes and pretreated with K7G and tilianin (2.5, 5, 10 and 20 µM, each); after 1 h, the cells were stimulated using α-MSH (200 nM) and then incubated for the next hour. The cells were treated with lysis buffer containing protease inhibitors (50 mM Tris-HCl, pH 7.4), 150 mM NaCl, 1 mM EDTA, 0.5% NP-40, 0.1% SDS, 1 mM EGTA, 100 μg/mL PMSF, 10 μg/mL pepstatin A, and 100 μM Na_3_VO_3_). The homogenates were centrifuged at 14,000 rpm and 4 °C for 15 min, and the protein concentrations in the supernatants were determined using Bradford reagent (Bio-Rad, Hercules, CA, USA). The total proteins (20 μg) in the supernatants were separated by SDS-PAGE at 100 V for 90 min on a 10% gel and blotted to a PVDF (Millipore Corporation, Billerica, MA, USA) membrane. The blotted membrane was incubated with a blocking solution (5% skim milk), followed by overnight incubation at 4 °C with an appropriate primary antibody. The following primary antibodies and dilutions were used: anti-tyrosinase (Santa Cruz Biotechnology, Santa Cruz, CA, USA), anti-TRP-1 (Santa Cruz Biotechnology), anti-MITF (Santa Cruz Biotechnology), anti-p-ERK, ERK (Santa Cruz Biotechnology), anti-p-JNK, JNK (Santa Cruz Biotechnology), anti-p-p38, p38 (Cell Signaling Technology, Beverly, MA, USA), anti-pCREB, CREB (Cell Signaling), anti-α-tubulin (Millipore, Billerica, CA, USA) and anti-β-actin (Cell Signaling). The membranes were washed three times with a Tris-buffered saline solution containing 0.1% Tween 20 (TBST) and then incubated with a horseradish peroxidase (HRP)-conjugated secondary antibody (Santa Cruz Biotechnology) for 1 h at room temperature. The membranes were washed three times with TBST and visualized with an enhanced chemiluminescence (ECL) kit (Thermo, Carlsbad, CA, USA). For quantitative analysis, the densitometric band values were determined by a bio-imaging analyzer (LAS-4000 mini, Fujifilm, Tokyo, Japan).

## 4. Conclusions 

K7G and tilianin along with 11 other compounds were isolated from *C. japonica*. The melanin content and cell viability of these compounds were determined using murine B16 cells. K7G and tilianin had inhibitory effects on melanogenesis. To understand the mechanism of action of the compounds, we examined several key factors related to the signaling pathways of melanogenesis in melanoma cells. K7G and tilianin significantly inhibited MITF, tyrosinase and TRP-1 protein expression in murine B16 cells. Moreover, CREB phosphorylation was suppressed by these two compounds. Our results also demonstrated that K7G and tilianin enhanced the phosphorylation of ERK. Hereafter, the pharmacological effects of *C. japonica* along with K7G and tilianin should be performed in various animal models to develop an anti-melanogenic drug.

## Figures and Tables

**Table 1:** Melanin content of the constituents isolated from *C. japonica* with an inhibitory effect on melanogenesis in murine B16 melanoma cells stimulated by α-MSH and their cell viability.

Sample	Cont. (μM)	Melanin Content (%)	Cell Viability	Sample	Cont. (μM)	Melanin Content (%)	Cell Viability
Vanillic acid	5	98.03	97.5	Tilianin	5	**108.55**	105.3
	10	104.27	98.6		10	**86.01**	101.2
	20	103.02	99.3		20	**32.64**	102.5
Chlorogenic acid	5	119.69	97.2	Apigetrin	5	130.83	78.8
	10	108.55	97.5		10	157.25	95.6
	20	60.1	101.6		20	182.12	97.7
3-*O*-Feruloylqunic acid	5	93.47	92.2	Wogoniside	5	96.03	105.3
	10	107.79	93.5		10	112.2	97.9
	20	108.79	97.7		20	106.05	94.5
Kaempferol-7-*O*-β-d-glucuronide	5	**104.87**	105.3	Naringenin	5	117.2	96.6
	10	**66.67**	97.9		10	106.5	99.8
	20	**23.08**	94.5		20	98.5	95.6
Acacetin-7-*O*-β-d-glucuronide	5	198.46	96.6	Genistein	5	121.2	64.5
	10	250.26	99.8		10	108.3	95.4
	20	24.62	95.6		20	115.6	95.3
Acacetin	5	106.2	90.4	β-sitosterol	5	105.65	64.5
	10	115.3	78.5		10	98.23	95.6
	20	109.3	90.6		20	101.57	97.2
Diosmetin	5	111.02	93.8	Arbutin	200	29.14	93.8
	10	106.08	99.8	a-MSH	+	100	
	20	103.58	95.6		-		100

“+” is denoted as α-MSH treated group; “-” is denoted as α-MSH untreated group.
